# China's engagement in global health governance: A critical analysis of China's assistance to the health sector of Africa

**DOI:** 10.7189/jogh.04.010301

**Published:** 2014-06

**Authors:** Xiangcheng Wang, Tao Sun

**Affiliations:** Zhou Enlai School of Government, Nankai University, Tianjin, China

**Figure:**
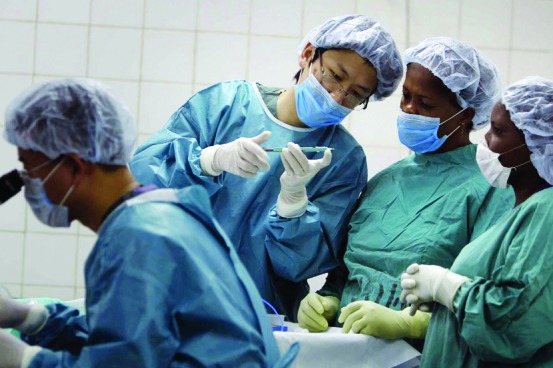
Photo: Courtesy of Xiangcheng Wang, personal collection

In recent years, China has been getting increasingly involved in global health governance. As the largest developing country and the second largest economy in the world, China’s engagement in global health governance has drawn considerable attention in the rest of the world. In this viewpoint, China’s medical and health–related assistance to Africa has been used as a case study to analyse China’s involvement in global health governance.

This paper will first assess China's motives and reasons for providing medical assistance to the health sector of Africa; then it will examine China's contributions and achievements in medical assistance. It will explore the major problems identified in China's medical assistance so far, and finally, it will draw conclusions based on China’s only official white paper on its foreign aid. To achieve those aims, this paper will use academic articles and books, government official documents and data, working papers from non–state organizations and think tanks related to China's medical assistance to Africa.

## Motives for China's medical assistance to Africa

The history of China's official medical assistance to Africa spans over half a century. China's government dispatched medical teams to Algeria in the January of 1963, which was historically the first official Chinese medical assistance to Africa.

The motives for China to provide assistance to the health sector of Africa varied in different historical contexts. Initially, Chinese officials claimed that they offered medical assistance to Africa to improve health of African people and that their motives were primarily humanitarian. However, China's decision to provide medical assistance to Africa could also be seen as mainly driven by political considerations. Studies suggested that China's medical assistance to Africa was closely related to China's position in international geopolitical context and China's foreign policy–making [1,2]. From that point of consideration, some of the reasons for China's medical assistance to Africa may include: (i) competition with Taiwan for the seat in the United Nations, through winning support from African countries; (ii) exporting the ideology of socialist experience and proletarian internationalism; (iii) attempting to address challenges in the rapidly evolving international context, such as the sanctions from the western countries led by the US after the *June Fourth Incident* [2]. After the World War II and the establishment of the United Nations, Africa played an important role in the vote of international organizations, based on the rule of “one country–one vote”, which made it strategically important for China to maintain good relationships with Africa. To ensure the support from Africa, offering medical assistance was regarded as an effective method by the Chinese government.

Furthermore, economic benefits may have also been a motive for China to actively provide medical assistance to Africa in recent years, although this interpretation has been repeatedly denied by the Chinese officials. It could be argued that China's medical assistance is being used as one of the tools to pave the way for China's state–owned enterprises to win the market access in Africa. There, they could be interested in benefiting from the natural resources in countries with relatively weaker bargaining power. However, such interpretation has been denied by the State Council of China, who states that economic benefit may only be a potential outcome, rather than a motive [3].

## China's contributions and achievements in Africa

The forms of China’s medical assistance to Africa included: (i) dispatching medical teams, (ii) constructing health care facilities, (iii) providing medicines and medical equipment, and (iv) donating to health funds. In the past decades, China has dispatched medical teams consisting of more than 20000 medical professionals to 46 African countries and they had treated around 200 million African patients [1]. The African recipient countries and their starting year of receiving China's medical assistance are shown in **Table 1**.

**Table 1:** African recipient countries and starting year of receiving China's medical assistance

Country	Start	Country	Start
Algeria	1963	Gabon	1977
Zanzibar	1964	Gambia	1977
Somalia	1965	Benin	1978
Congo (Brazzaville)	1967	Zambia	1978
Mali	1968	Central African Republic	1978
Tanganyika (Tanzania)	1968	Chad	1978
Mauritania	1968	Botswana	1981
Guinea	1968	Djibouti	1981
Sudan	1971	Rwanda	1982
Equatorial Guinea	1971	Uganda	1983
Sierra Leone	1973	Libya	1983
Tunisia	1973	Cape Verde	1984
Democratic Republic of Congo	1973	Liberia	1984
Ethiopia	1974	Zimbabwe	1985
Togo	1974	Burundi	1986
Cameroon	1975	Seychelles	1987
Senegal	1975	Comoros	1994
Madagascar	1975	Namibia	1996
Morocco	1975	Lesotho	1997
Niger	1976	Eritrea	1997
Mozambique	1976	Malawi	2008
Sao Tome and Principe	1976	Angola	2009
Guinea–Bissau	1976	Ghana	2009

* Source: Adapted from Li [1].

## Some possible concerns over China's medical assistance to Africa

There are some possible concerns over the content and the process of China's medical assistance to Africa, including: (i) the relatively limited financial commitment; (ii) the controversy over the effectiveness of traditional Chinese medicine (TCM); (iii) the controversy over selection criteria of the deployed medical professionals; and (iv) the assistance not being proportional to the national strength.

Considering that China is still a developing country, its total contribution to the health sector and health care–related projects of Africa could be viewed as substantial. The average annual investment in hospitals and disease control centers amounted to around US$ 8 million and US$ 500000, respectively. In addition, the average annual cost of medical teams and required medicines are US$ 14 million and US$ 60 million, respectively [2]. Moreover, China has also made a commitment to donate US$ 14 million before 2013 to the Global Fund, of which the major beneficiaries are African countries. Therefore, the total cost of China's medical assistance to Africa has amounted to at least US$ 80 million in each year since 2007.

However, from the perspective of China's emerging role as the 2nd largest economy in the world, the actual amount of its medical assistance to Africa is relatively limited in comparison to other donor nations and non–governmental organizations (NGOs). The average amount of China's spending on health–related assistance to each African country that it supports is only around US$ 1.8 million per year. As an example, **Table 2** shows the amount of support that some donor nations and NGOs commit to some of the recipient countries:

**Table 2:** The amount of financial support that some donor nations and international organizations provided to selected recipient countries

Donor nation or organization	Recipient country	Year	Amount	Aim
France	Kenya	2010	€ 35.5 million	To strengthen the capacity of health care division
Belgium	Rwanda	2011	€ 55 million	To assist the medical and health–related needs
European Union	DRC	2010	€ 51 million	To improve its overall level of health services by supporting its public health development plan and improving its essential medicine supply system
World Bank	Southern Sudan (now the Republic of South Sudan)	2010	US$ 63 million	To develop its health system and to improve its overall medical and health care conditions
Global Fund to Fight AIDS, Tuberculosis and Malaria	Zambia	2012	US$ 100 million	To scale up prevention and strengthen its health systems
United States	Burundi	2012	US$ 8.3 million	To assist the medical and health–related needs
United States	Kenya	2012	US$ 545 million	To develop the disease control ability and to improve the health system
United States	Algeria	2012	US$ 471 million	Same as above
United States	Tanzania	2012	US$ 436 million	Same as above
United States	Uganda	2012	US$ 323 million	Same as above

*Source: French Development Agency [4], Belgium Development Agency [5], European Commission [6], World Bank [7], The Global Fund [8], US Government [9].

Another somewhat controversial issue is that of traditional Chinese medicine (TCM), including herbal medicine and acupuncture therapy. TCM is widely used in China’s medical assistance to African countries, although its effectiveness is a matter of much debate internationally. The TCM lacks the support from double–blind randomized controlled trials, which are recognized by the modern medical science as the best way to evaluate treatments; until those trials have been conducted, the placebo effect will remain a possible explanation for much of the observed efficacy of the TCM [10].

## Findings after analyzing *China’s Foreign Aid* document

Through analyzing *China’s Foreign Aid*, China's first and only official document on its foreign aid so far, several findings could be proposed.

First, when providing medical assistance to Africa, China is affected by political interests. China's government claims that China would not impose any political conditions to the recipient countries. However, in fact, China suspended all the medical assistance and cooperation, and withdrew all the medical teams in the African recipient countries which respectively built formal diplomatic relationships with Taiwan during 1980s and 1990s, until those countries stopped recognized Taiwan's political status. In those cases, China's medical assistance was related to its interference of the recipient countries' own decisions on the political interactions with Taiwan.

Second, China’s foreign aid has been distributed mainly through three types of financing: free grants, interest–free loans, and concessional loans. The total amount of China’s financial resources committed to foreign aid has become substantial – China has pledged ￥ 256.29 billion in foreign aid by 2009; however, the amount for medical and health–related assistance seems to account for only a small part of that large budget. Only around 6% of free grants and 3% of the loans are used in the public facilities in Africa, in which the hospital, medical station and other health facilities take even smaller portion and receive fewer financial resources. Besides that, loans may not be very suitable for some African countries that are facing economic challenges and lack capacity to repay those loans.

Third, as stated in the document, the forms of China’s foreign aid include: complete projects, general goods and materials, technical cooperation, human resources, development cooperation, foreign aid medical teams, emergency humanitarian assistance, overseas volunteer programs, and debt relief. Some forms, including foreign aid medical teams, general goods and materials, as well as debt relief, have evidently made contributions and played positive roles in China’s medical assistance to Africa to a considerable degree; while the other forms of foreign aid did not focus on health–related issues, thus having little impact on Africa’s health care.

Fourth, in terms of geographical distribution, African countries are the major beneficiary countries of China’s foreign aid; while in terms of the distribution of major areas of development aid, it is evident that the assistance to the health care sector has not been the key focus of China’s foreign aid, since the projects established in the health care sector are proportionally the smallest among all other areas of aid and the history of the foreign aid to the health care is shorter than that of any other area.

Fifthly, in terms of management of foreign aid, the State Council has nominally the leadership and management rights over China's foreign aid. However, unlike most OECD member states, which have established independent and specialized agencies for foreign aid (eg, USAID, DFID, AUSAID, NORAD and SIDA), in China, numerous ministries, departments and bureaus are authorized to partly deal with, or to be responsible for the affairs of assistance – including medical aid to Africa. There are overlapping functions between those ministries and departments and conflicts are likely to occur in the process of implementation. This reduces the efficiency of provided assistance and likely increases the cost of medical assistance. China does not have an independent and specialized agency for foreign aid, and the current foreign aid management cannot be considered effective, open, or transparent enough.

Sixthly, in terms of international cooperation, China has been trying to strengthen multilateral and regional cooperation with other aid–providers, such as the Global Fund and the Bill & Melinda Gates Foundation, to provide medical assistance to Africa since 2005.

## Conclusion

Through examining and analyzing the existing literature on China’s medical assistance to Africa, as well as conducting a critical analysis of China’s only official white paper on its foreign aid, a number of key findings have been proposed. First, China’s medical assistance to Africa has a history of 60 years, and has made notable contributions to the health care sector of Africa. Second, Africa is the major beneficiary region of China’s foreign aid, but health care is not the most important component of China’s foreign aid. Third, the forms of China’s foreign aid vary and some of them indeed make contributions to Africa’s health care to a different degree, while the others do not. Fourthly, the management of China’s medical assistance to Africa is not effective and open enough, and China’s medical aid work is not always operating coherently and properly.

Some implications could be drawn from the case study of China’s medical aid to Africa for China’s overall involvement in global health governance. For instance, China could attach more importance to global health governance, and could learn from the US “Global Health Initiative” and UK’s “Health is Global” strategy to formulate and develop a clear, coherent and well–designed strategy or plan for its involvement in global health governance. Besides, solving domestic health care challenges would be beneficial for China's involvement in global health governance, where it could lead by example in more appropriately utilizing the limited health care resources. Moreover, China could explore opportunities to enhance international and multilateral cooperation with other actors to become more comprehensively involved in global health governance.
